# ERGA-BGE reference genome of *Eunicella cavolini, *an IUCN Near Threatened Gorgonian of the Mediterranean Sea

**DOI:** 10.12688/openreseurope.21514.1

**Published:** 2025-10-22

**Authors:** Didier Aurelle, Dorian Guillemain, Frédéric Zuberer, Denys Malengros, Astrid Böhne, Rita Monteiro, Thomas Marcussen, Torsten H. Struck, Rebekah A. Oomen, Genoscope Sequencing Team, Alice Moussy, Corinne Cruaud, Karine Labadie, Lola Demirdjian, Caroline Belser, Patrick Wincker, Pedro H. Oliveira, Jean-Marc Aury, Chiara Bortoluzzi

**Affiliations:** 1 Université de Toulon, CNRS, IRD, MIO, Aix Marseille Universite, Marseille, 13009, France; 2 Institut Systématique Evolution Biodiversité (ISYEB), CNRS, Sorbonne Université, EPHE, Université des Antilles, CP 26, Muséum national d’Histoire naturelle, Paris, 75005, France; 3 CNRS, IRD, IRSTEA, OSU PYTHEAS, Aix Marseille Univer, Marseille, France; 4 Leibniz Institute for the Analysis of Biodiversity Change, Museum Koenig Bonn, Bonn, 53113, Germany; 5 University of Oslo, Natural History Museum, Oslo, Norway; 6 University of Oslo, Centre for Ecological & Evolutionary Synthesis, Oslo, Norway; 7 Department of Biological Sciences, University of New Brunswick Saint John, Saint John, Canada; 8 University of Gothenburg, Tjärnö Marine Laboratory, Gothenburg, Sweden; 9 University of Agder, Centre for Coastal Research, Kristiansand, Norway; 10 Institut François Jacob, CEA, CNRS, Univ Evry, Université Paris-Saclay, Genoscope, Evry, 91057, France; 11 Institut François Jacob, CEA, CNRS, Univ Evry, Université Paris-Saclaye, Génomique Métabolique, Genoscope, Evry, 91057, France; 12 SIB Swiss Institute of Bioinformatics, Amphipôle, Quartier UNIL-Sorge, Lausanne, 1015, Switzerland

**Keywords:** Eunicella cavolini, genome assembly, European Reference Genome Atlas, Biodiversity Genomics Europe, Earth Biogenome Project, Eunicellidae, yellow gorgonian

## Abstract

The *Eunicella cavolini* reference genome provides an important resource to study the adaptation of this species to different environments and anthropic pressures. This species is impacted by human activities, including climate change, and this reference genome will be useful to study the genomic evolution of this species. The entirety of the genome sequence was assembled into 17 contiguous chromosomal pseudomolecules. This chromosome-level assembly encompasses 0.49 Gb, composed of 159 contigs and 46 scaffolds, with contig and scaffold N50 values of 7.7 Mb and 51.1 Mb, respectively.

## Introduction

*Eunicella cavolini*, also known by the French local name of Gorgone jaune, is a cnidaria member of the sub-class Octocorallia and of the Eunicellidae family ( McFadden *et al.*, 2022) and is one of the most common gorgonians in the Mediterranean Sea. This species can be found in the Western and Eastern Mediterranean Sea, at depths of less than 10 meters and more than 150 meters ( Carugati *et al.*, 2022; Sini *et al.*, 2015). *Eunicella cavolini* is currently classified as 'Near Threatened' on the IUCN Red List ( Otero *et al.*, 2017). As other Mediterranean octocorals, *Eunicella* species are impacted by mass mortality events linked with marine heat waves, fishing activities, or pollution ( Garrabou *et al.*, 2022; Sini *et al.*, 2015; Topçu & Öztürk, 2015). Along with other gorgonians *, E. cavolini* plays an important ecological role by creating 3D habitats supporting a diversity of species ( Gibson *et al.*, 2006), including various bacterial species depending on the environment. Nonetheless, the dominant bacterial taxa are from the genus *Endozoicomonas* (Gammaproteobacteri) ( Bayer *et al.*, 2013). A high-quality reference genome for *E. cavolini* is useful to study the genomic diversity of this species and its adaptive potential facing contrasted and changing environments ( Aurelle *et al.*, 2022). Such genomic resources are pivotal for species management, allowing, for example, the identification of key populations to be conserved. The genome will also be used to study species limits and hybridization with other *Eunicella* species ( Aurelle *et al.*, 2024). 

The generation of this reference resource was coordinated by the European Reference Genome Atlas (ERGA) initiative’s Biodiversity Genomics Europe (BGE) project, supporting ERGA’s aims of promoting transnational cooperation to promote advances in the application of genomics technologies to protect and restore biodiversity ( Mazzoni *et al.*, 2023).

## Materials & methods

ERGA's sequencing strategy includes Oxford Nanopore Technology (ONT) and/or Pacific Biosciences (PacBio) for long-read sequencing, along with Hi-C sequencing for chromosomal architecture, Illumina Paired-End (PE) for polishing (i.e. recommended for ONT-only assemblies), and RNA sequencing for transcriptomic profiling, to facilitate genome assembly and annotation.

### Sample and sampling information

Dorian Guillemain, Frédéric Zuberer and Denys Malengros, all from the Pythéas Institute, sampled one specimen of *Eunicella cavolini* (sex unknown), which was identified based on macro-morphology and colour. The identification was performed by Dorian Guillemain, Frédéric Zuberer and Didier Aurelle. The sample was collected on the Frioul archipelago, Marseille, France, on the 19th of July 2023. Sampling was performed under permission Arrêté n°107 issued by the Direction Interrégionale de la mer Méditerranée. Sampling was performed through scuba diving. The specimen was euthanized by being put flash frozen at -80 °C. The sample was preserved at this temperature until DNA and RNA extraction. 

### Vouchering information

Physical reference materials for the here sequenced specimen have been deposited in the Museum National d'Histoire Naturelle, Paris ( https://www.mnhn.fr/fr) under the accession number MNHN-IK-2019-2701.

Frozen reference tissue material from the same individual is being deposited at the Museum National d'Histoire Naturelle, Paris https://www.mnhn.fr/fr.

### Genetic information

The estimated genome size, estimated by Genomes on a Tree (GoaT) ( Challis *et al.*, 2023) by ancestral state reconstruction, is 0.62 Gb. This is a diploid genome with a haploid number of 6 chromosomes (2n=12). All information for this species was retrieved from GoaT.

### DNA/RNA processing

DNA extraction was performed by first grinding 400 mg of reproductive tissue in liquid nitrogen, followed by digestion in 5 mL of buffer containing 30 mM Tris-HCl, 10 mM EDTA, 1% SDS, and proteinase K (20 µL/mL) at 53°C for 4 h. The lysate was centrifuged at 500 × g for 5 min at room temperature to remove debris, including sclerites. To the resulting supernatant, 10 mL of absolute ethanol were added, and the mixture was incubated at −20 °C for 1 h. DNA was pelleted by centrifugation at 8500 × g for 15 min at 4 °C, washed once with 70% ethanol, and centrifuged again under the same conditions. The pellet was then resuspended in 9.5 mL of G2 buffer from Genomic Tip 100/G kit (QIAGEN, MD, USA) and incubated overnight at 4 °C. The following day, RNase A (19 µL; 100 mg/mL) was added and the sample was incubated for 1 h at 50 °C. DNA was subsequently purified using the Genomic Tip 100/G kit according to the manufacturer’s protocol. DNA fragment size selection was performed using Short Read Eliminator (PacBio). Quantification was performed using a Qubit dsDNA HS Assay kit (Thermo Fisher Scientific) and integrity was assessed in a FemtoPulse system (Agilent). DNA was stored at 4 ºC until usage.

RNA was extracted from reproductive tissue (70 mg) using RNeasy Plus Universal Kit (Qiagen) following manufacturer instructions. Residual genomic DNA was removed with 6U of TURBO DNase (2 U/μL) (Thermo Fisher Scientific). Quantification was performed using a Qubit RNA HS Assay and integrity was assessed in a Bioanalyzer system (Agilent). RNA was stored at -80°

### Library preparation and sequencing

Long-read DNA library was prepared with SMRTbell prep kit 3.0 following manufacturers' instructions and sequenced on a Revio system (PacBio). Hi-C library was generated from reproductive tissue using the Arima High Coverage HiC Kit (following the Animal Tissues low input protocol v01) and sequenced on a NovaSeq6000 instrument (Illumina) with 2 x 150 read length. Poly(A) RNA-Seq libraries were constructed using the Illumina Stranded mRNA Prep, Ligation Prep kit (Illumina) and sequenced on a Illumina NovaSeq X Plus instrument.

In total 53x PacBio and 130x HiC data were sequenced to generate the assembly.

### Genome assembly methods

The genome of *Eunicella cavolini* was assembled using the Genoscope GALOP pipeline ( https://workflowhub.eu/workflows/1200). Briefly, raw PacBio HiFi reads were assembled using Hifiasm v0.19.8-r603 ( Cheng *et al.*, 2021). Remaining allelic duplications were removed using purge_dups v1.2.5 ( Guan *et al.*, 2020) with default parameters and the proposed cutoffs, but all contigs larger than 1Mb were kept in the purged assembly. This assembly was scaffolded using YaHS v1.2.2 ( Zhou *et al.*, 2023) and assembled scaffolds were then curated through manual inspection using PretextView v0.2.5 to remove false joins and incorporate sequences not automatically scaffolded into their respective locations within the chromosomal pseudomolecules. Chromosome-scale scaffolds confirmed by Hi-C data were named in order of size. The mitochondrial genome was assembled as one circular contig using Oatk v1.0 ( Zhou *et al.*, 2025) and included in the released assembly. Summary analysis of the released assembly was performed using the ERGA-BGE Genome Report ASM Galaxy workflow ( 10.48546/workflowhub.workflow.1104.1).

## Results

### Genome assembly

**Figure 1.**
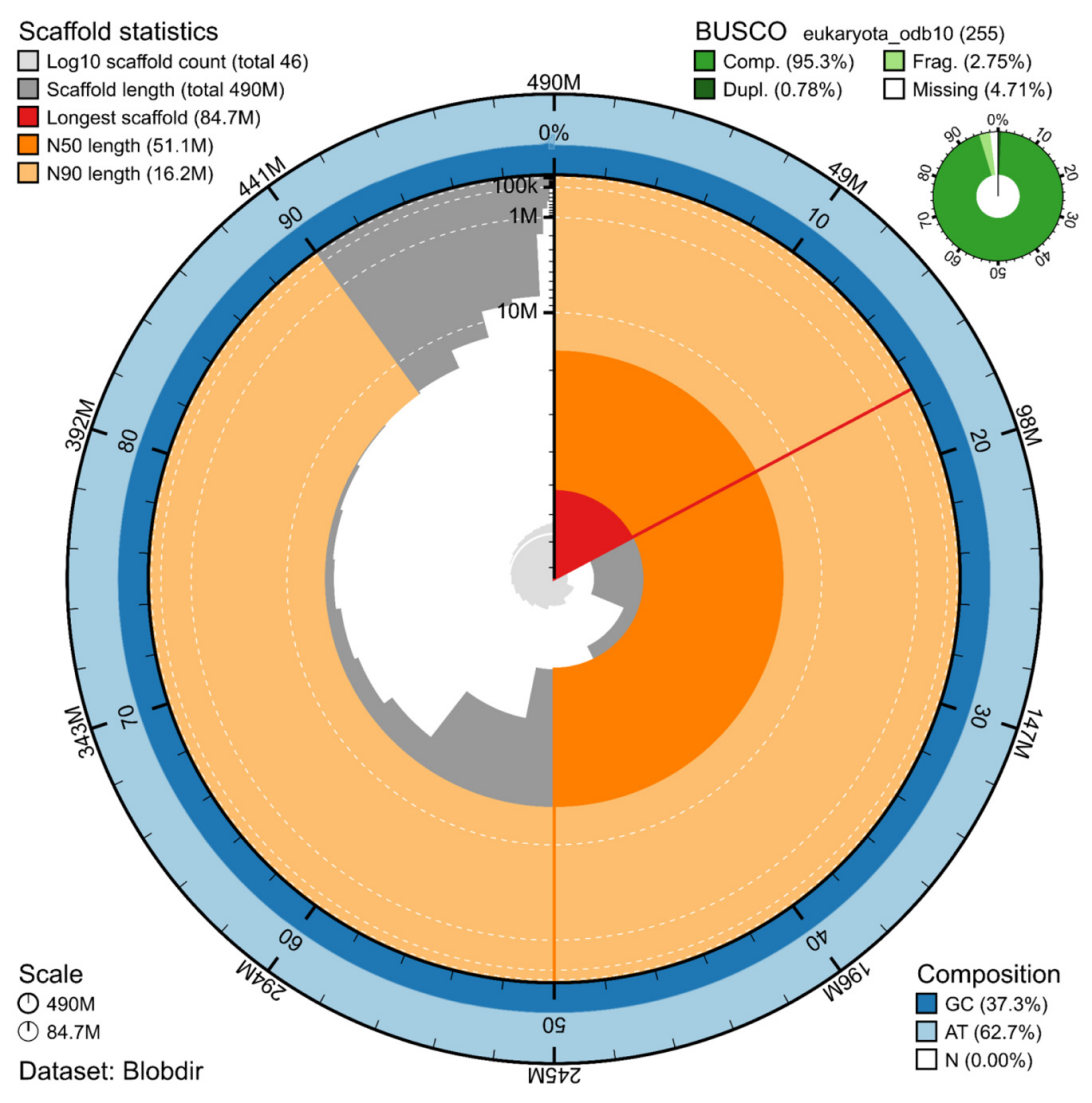
Snail plot summary of assembly statistics. The main plot is divided into 1,000 size-ordered bins around the circumference, with each bin representing 0.1% of the 489,969,558 bp assembly. The distribution of sequence lengths is shown in dark grey, with the plot radius scaled to the longest sequence present in the assembly (84.7 Mb, shown in red). Orange and pale-orange arcs show the scaffold N50 and N90 sequence lengths (51,102,291 and 16,173,997 bp), respectively. The pale grey spiral shows the cumulative sequence count on a log-scale, with white scale lines showing successive orders of magnitude. The blue and pale-blue area around the outside of the plot shows the distribution of GC, AT, and N percentages in the same bins as the inner plot. A summary of complete, fragmented, duplicated, and missing BUSCO genes found in the assembled genome from the Eukaryota database (odb10) is shown in the top right.

**Figure 2.**
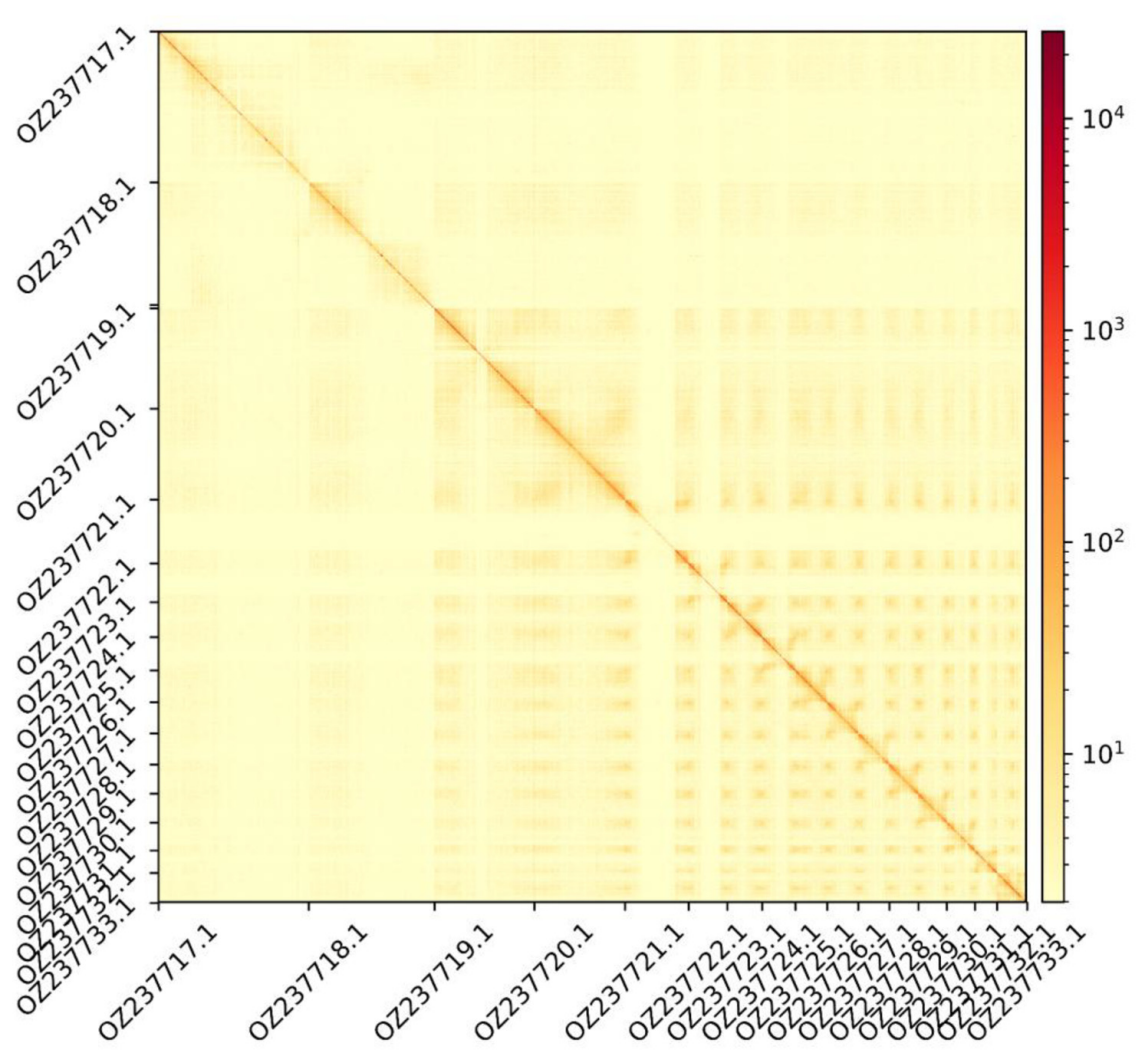
Hi-C contact map showing spatial interactions between regions of the genome. The diagonal corresponds to intra-chromosomal contacts, depicting chromosome boundaries. The frequency of contacts is shown on a logarithmic heatmap scale. Hi-C matrix bins were merged into a 100 kb bin size for plotting.

The genome assembly has a total length of 489,969,558 bp in 46 scaffolds ( Figure 1 & Figure 2), with a GC content of 37.3%. The assembly has a contig N50 of 7,689,143 bp and L50 of 20 and a scaffold N50 of 51,102,291 bp and L50 of 4. The assembly has a total of 113 gaps, totalling 15.3 kb in cumulative size. The single-copy gene content analysis using the Eukaryota database with BUSCO ( Manni *et al.*, 2021) resulted in 95.3% completeness (94.5% single and 0.8% duplicated). 80.6% of reads k-mers were present in the assembly and the assembly has a base accuracy Quality Value (QV) of 62.4 as calculated by Merqury ( Rhie *et al.*, 2020).

## Data Availability

*Eunicella cavolini* and the related genomic study were assigned to Tree of Life ID (ToLID) 'jaEunCavo1' and all sample, sequence, and assembly information are available under the umbrella BioProject PRJEB79972. The sample information is available at the following BioSample accession SAMEA115358980. The genome assembly is accessible from ENA under accession number GCA_965177985. 1. The annotated genome will be made available through the Ensembl website ( https://projects.ensembl.org/erga-bge/). Sequencing data produced as part of this project are available from ENA at the following accessions: ERX14096372, ERX14096373, ERX14096374, ERX14096395, ERX14096397, ERX14169049, and ERX14169050. Documentation related to the genome assembly and curation can be found in the ERGA Assembly Report (EAR) document available at https://github.com/ERGA-consortium/EARs/tree/main/Assembly_Reports/Eunicella_cavolini/jaEunCavo1. Further details and data about the project are hosted on the ERGA portal at https://portal.erga-biodiversity.eu/data_portal/317547.

## Author information

Members of the Genoscope Sequencing Team are listed here: https://zenodo.org/records/14611490.
