# Supplementary material for: ERGA-BGE reference genome of Eunicella cavolini, an IUCN Near Threatened Gorgonian of the Mediterranean Sea
Source: Open Res Eur. 2026 Jul 27;5:323. Originally published 2025 Oct 22. [Version 2] doi: 10.12688/openreseurope.21514.2 (PMC12759274; doi:10.12688/openreseurope.21514.2)
Supplement: Peer review history

# Reviewer report: Reviewer response for version 2

**DOI:** 10.21956/openreseurope.25369.r78106

**Published:** 2026-07-28

**Authors:** Alan Elena

**Affiliations:** 1 Technische Universität (TU), Dresden, Germany

I thank the authors for the responses to my comments. I have nothing more to add.

Are sufficient details of methods and materials provided to allow replication by others?

Yes

Is the rationale for creating the dataset(s) clearly described?

Yes

Are the datasets clearly presented in a useable and accessible format?

Yes

Are the protocols appropriate and is the work technically sound?

Yes

Reviewer Expertise:

Bioinformatics, genomics, microbiology, environment, clinic.

I confirm that I have read this submission and believe that I have an appropriate level of expertise to confirm that it is of an acceptable scientific standard.

# Reviewer report: Reviewer response for version 1

**DOI:** 10.21956/openreseurope.23272.r66318

**Published:** 2026-01-02

**Authors:** Alan Elena

**Affiliations:** 1 Technische Universität (TU), Dresden, Germany

Aurelle *et al. *describe the generation of the only available *E. cavolini* genome. The assembly quality is high enough to be classified as a reference sequence. The provided data is relevant since it provides a high quality benchmark for future comparison. Authors highlight the relevance of such sequences in a proper way. I believe the communication provides what it is supposed to provide and there are no major comments on my side.

 Just a few minor questions:

 1) Was this analysis done only once on one individual? Maybe to minimise genetic variability in a reference sequence and ensure robustness of results, this protocol could be repeated on independent organisms.

 2) Given the fragmentation of the genome and the status of this entry as RefSeq, was there/will there be an attempt to close the scaffolds?

 3) Why was reproductive tissue selected for sequencing?

 4) How did the authors deal with  potential DNA contamination from colonising species?

 5) Aside from the present, there are very few sequences belonging to this species and most of these focus on specific structures (e.g.: Cytochromes) rather than on broader fragments of DNA. What is the reason for this?

Are sufficient details of methods and materials provided to allow replication by others?

Yes

Is the rationale for creating the dataset(s) clearly described?

Yes

Are the datasets clearly presented in a useable and accessible format?

Yes

Are the protocols appropriate and is the work technically sound?

Yes

Reviewer Expertise:

Bioinformatics, genomics, microbiology, environment, clinic.

I confirm that I have read this submission and believe that I have an appropriate level of expertise to confirm that it is of an acceptable scientific standard.

# Response

**Published:** 2026-03-13

**Authors:** Chiara Bortoluzzi

**Affiliations:** Swiss Institute of Bioinformatics, Lausanne, Vaud, Switzerland

We thank the reviewer for their enthusiasm regarding this Genome Report and production of the E. cavolini genome. We have addressed their comments as below: 

1. The analysis was done only once and it cannot be repeated as there is no additional material available for the species and funding is also finished due to the end of the BGE project that funded this genome assembly;
2. This is a chromosome-level assembly and is not that fragmented as indicated by its contig N50 and scaffold N50. The genome assembly passed the ERGA Assembly Report, which is also meant to flag any potential fragmentation issues. None were found;
3. The use of the reproductive tissue (now referred to as coenenchyme) is a standard procedure for octocorals as it is difficult to separate specific tissues;
4. There was no evidence of macro-organism at the surface of the colony. Therefore, the risk of DNA contamination was very low. This is also confirmed by the contamination analysis reported in the ERGA Assembly Report: https://github.com/ERGA-consortium/EARs/blob/main/Assembly_Reports/Eunicella_cavolini/jaEunCavo1/jaEunCavo1.1_EAR.pdf;
5. The genetic diversity and genetic structure of this species had previously been studied with microsatellites (Masmoudi et al., 2016), and more recently with transcriptome and RAD sequencing data (Romiguier et al., 2014; Aurelle et al., 2024).

References: Masmoudi, M. B., Chaoui, L., Topçu, N. E., Hammami, P., Kara, M. H., & Aurelle, D. (2016). Contrasted levels of genetic diversity in a benthic Mediterranean octocoral: Consequences of different demographic histories?. Ecology and Evolution, 6(24), 8665-8678. Romiguier, J., Gayral, P., Ballenghien, M., Bernard, A., Cahais, V., Chenuil, A., ... & Galtier, N. (2014). Comparative population genomics in animals uncovers the determinants of genetic diversity. Nature, 515(7526), 261-263. Aurelle, D., Haguenauer, A., Bally, M., Zuberer, F., Guillemain, D., Ledoux, J. B., ... & Pontarotti, P. (2024). Symbiosis, hybridization, and speciation in Mediterranean octocorals (Octocorallia, Eunicellidae). Biological Journal of the Linnean Society, 143(4), blae116.

# Reviewer report: Reviewer response for version 1

**DOI:** 10.21956/openreseurope.23272.r62849

**Published:** 2025-11-05

**Authors:** Benjamin D. Young

**Affiliations:** 1 University of Colorado Boulder Department of Ecology and Evolutionary Biology, Boulder, Colorado, USA

This is a fantastic resource for the soft coral community and the assembly pipeline follows best practices. I have a few small comments that I believe will improve the manuscript readability and should be super easy to incorporate!

- *Along with other gorgonians, E. cavolini plays an important ecological role by creating 3D habitats supporting a diversity of species (* *Gibson et al., 2006), including various bacterial species depending on the environment. Nonetheless, the dominant bacterial taxa are from the genus Endozoicomonas (Gammaproteobacteri) ( Bayer et al., 2013).*

This sentence reads a little weird to me, especially as the *Endozoicomonas* are within the coral microbiome form the Bayer paper and the statement you are making seems to be about the habitat the coral generates. I would recommend rewording this section or removing the section about the bacteria and microbiome as I do not think it is needed.

- *The specimen was euthanized by being put flash frozen at -80 °C.*

Remove the “put” it makes the sentence read weird.

 You say “reproductive tissue” a few times, can you be a little more specific? I understand the sex was unknown so did you just grind up several whole polyps? Or did you extract mesenteries form within the polyps?

- *In total 53x PacBio and 130x HiC data were sequenced to generate the assembly.*

This is wonderful but did you get any results from the RNA-sequencing? You do specify you did RNA-prep and sequencing in the methods but then there is no further mention anywhere in the report of why you did this or what it was used for. I assume it was/is being used for annotation so maybe stating that would be good, and potentially providing some preliminary results for the gene prediction and annotation? If not, then I would recommend removing the RNA aspects completely.

- *and assembled scaffolds were then curated through manual inspection using PretextView v0.2.5 to remove false joins and incorporate sequences not automatically scaffolded into their respective locations within the chromosomal pseudomolecules.*

Is there an article/github citation you can include for PretextView ?

- *The genome assembly has a total length of 489,969,558 bp in 46 scaffolds (* *Figure 1 & Figure 2), with a GC content of 37.3%. The assembly has a contig N50 of 7,689,143 bp and L50 of 20 and a scaffold N50 of 51,102,291 bp and L50 of 4.*

This is a little nit-picky, but can you add in at the beginning the results that these results you are stating are for the 17 contiguous chromosomal pseudomolecules? It just makes it instantly obvious which assembly (initial versus chromosomal) you are talking about. I had to scroll to the abstract to make sure this was the chromosomal stats.

 This is not necessary but could be nice, before giving the chromosomal results could you give the initial assembly size/stats from just HiFiAsm? I think it is interesting to see how use of HiC can improve the assembly. Again, not necessary at all if this is too much extra work!

- *The single-copy gene content analysis using the Eukaryota database with BUSCO *

Can you please include the version (i.e. odb10, odb12) BUSCO database you used. 

 Again, this is wonderful work and a amazing resource for researchers using this species!

Are sufficient details of methods and materials provided to allow replication by others?

Yes

Is the rationale for creating the dataset(s) clearly described?

Yes

Are the datasets clearly presented in a useable and accessible format?

Yes

Are the protocols appropriate and is the work technically sound?

Yes

Reviewer Expertise:

I work in a number of different organisms (e.g., bacteria, fungi, coral, insects, fish) assembling genomes and undertaking genetic research.

I confirm that I have read this submission and believe that I have an appropriate level of expertise to confirm that it is of an acceptable scientific standard.

# Response

**Published:** 2026-03-13

**Authors:** Chiara Bortoluzzi

**Affiliations:** Swiss Institute of Bioinformatics, Lausanne, Vaud, Switzerland

We thank the reviewer for their enthusiasm regarding this Genome Report and production of the E. cavolini genome. We have addressed their comments as below: 

1. We removed the small paragraph in the introduction about the bacteria and microbiome;
2. We removed 'put' as suggested;
3. We decided to change the term 'reproductive tissue' with the term 'coenenchyme' as this is more appropriate for octocoral;
4. We did produce RNA-Seq data, which are available at the following BioProject: PRJEB79972. The RNA-Seq data were used for the annotation, which is available in Ensembl. We added a new section about the genome annotation for completeness. 
5. We added the citation to PretextView as suggested.
6. We added a new sentence at the beginning of the results section as suggested;
7. We added the version of the BUSCO database used. 
